# Clinical Study on the Application of Preserved Urethral Mucosa at the Prostatic Apex in Transurethral Plasmakinetic Resection of the Prostate

**DOI:** 10.3389/fsurg.2022.922479

**Published:** 2022-06-17

**Authors:** Jun-Qiang Liang, Wang-Teng Ma, Bin-Wei Lu, Liang Dai, Yu-Ming Zhao, Ji-Dong Zhang, Bao Tian, Qing-Li Liu

**Affiliations:** ^1^ Department of Urology, Hebei North University, Zhangjiakou, China; ^2^ Department of Urology, First Hospital of Qinhuangdao, Qinhuangdao, China; ^3^ Department of Surgery, Hebei Medical University, Shijiazhuang, China

**Keywords:** benign prostatic hyperplasia, urethral mucosa, external urethral sphincter, transurethral plasmakinetic resection of the prostate, urinary incontinence

## Abstract

**Objective:**

To explore the differences in the clinical efficacy, complications, and safety of transurethral plasmakinetic resection of the prostate (PKRP) by the conventional approach versus the approach preserving the urethral mucosa at the prostatic apex in the treatment of benign prostatic hyperplasia (BPH).

**Methods:**

A total of 90 patients with PKRP admitted to the First Hospital of Qinhuangdao from December 2018 to March 2021 were selected and divided into a control group (conventional PKRP, *n* = 45) and an observation group (PKRP with preserved urethral mucosa at the prostatic apex, *n* = 45). The clinical efficacy, safety, and sexual function of the groups were evaluated using the patients’ International Prostate Symptom Score (IPSS), quality of life (QoL), prostate volume, maximum flow rate (Qmax), post-void residual (PVR), blood loss, surgical resection efficiency, and surgical complication data.

**Results:**

The differences in the preoperative indicators, glandectomy quality, and glandectomy rate between the groups were not statistically significant (*P *> 0.05). However, in the observation group, the surgery time and blood loss were significantly lower compared with the control group, and the resection efficiency was significantly higher, with statistical significance (*P *< 0.05). In the follow-up, one month after surgery, the IPSS and QoL were lower in the observation group than in the control group, and the differences were statistically significant (*P *< 0.05); three months after surgery, the PVR, IPSS, QoL, and Qmax scores were similar between the groups, with no statistical significance (*P *> 0.05). In terms of surgical complications, the incidences of urinary incontinence and other complications after catheter extraction were significantly lower in the observation group than in the control group, and the differences between the groups were statistically significant (*P *< 0.05).

**Conclusion:**

Compared with conventional PKRP, PKRP with preserved urethral mucosa at the prostatic apex can lead to immediate urinary continence after catheter extraction, reduce intraoperative blood loss, and shorten the surgery time, thus improving the surgical efficiency.

## Introduction

Benign prostatic hyperplasia (BPH) is a disease caused by slow prostatic hyperplasia that eventually progresses to bladder outlet obstruction and associated lower urinary tract symptoms, seriously affecting patients’ quality of life (1). Surgery is still the most effective way to treat BPH (2). Transurethral resection of the prostate (TURP) was once considered the “gold standard” for BPH treatment (3, 4). However, a series of minimally invasive endovascular treatment methods have recently been derived (5), challenging the concept of TURP as the surgical benchmark. Plasmakinetic resection is becoming increasingly favored due to its few complications and good long-term effects (6, 7), yet there are still risks of postoperative urinary incontinence and other complications (8) that must be further optimized.

It is well known that open prostatectomy boasts a good surgical effect, resulting in almost no postoperative urinary incontinence (9). However, it has been found that part of the residual urethral mucosa of the prostate during open prostatectomy must sometimes be excised with scissors; the remaining urethral mucosa does not affect urination. Therefore, this study observes how preserving the urethral mucosa at the prostatic apex during TURP (10) leads to postoperative urinary continence and also examines the efficacy of plasmakinetic resection of the prostate (PKRP) with preserved urethral mucosa at the prostatic apex. The paper discusses the clinical efficacy and safety of the newly developed PKRP compared with conventional PKRP in BPH treatment.

## Materials and Methods

### Clinical Data

The clinical data of 90 patients undergoing plasmakinetic resection in our hospital from December 2018 to March 2021 were collected. Patients undergoing conventional PKRP were included in the control group (*n* = 45), and those undergoing PKRP with preserved urethral mucosa at the prostatic apex were included in the observation group (*n* = 45). All patients were carefully evaluated based on their digital rectal examination, B-ultrasonography, prostate-specific antigen, maximum flow rate (Qmax), post-void residual (PVR), International Prostate Symptom Score (IPSS), and quality of life (QoL) data and were diagnosed as having BPH, with clear indications for surgery. This study was approved by the ethics committee, and all patients signed the informed consent form. The preoperative indicators are shown in Table 1. The differences in the baseline data between the two groups were not statistically significant (*P *> 0.05).

**Table 1:** Preoperative indicators of patients in the two groups.

Groups	Age	PV/g	PVR/ml	Qmax	IPSS	Qol
Control group (*n* = 45)	68.60 ± 8.22	63.37 ± 20.74	59.71 ± 18.12	6.04 ± 1.66	24.78 ± 3.00	5.44 ± 0.50
Observation group (*n* = 45)	69.27 ± 6.15	67.61 ± 23.92	58.27 ± 19.43	6.10 ± 2.09	25.11 ± 2.81	5.38 ± 0.49
*t*-value	−0.436	−0.900	0.363	−0.146	−0.544	0.637
*P*-value	0.664	0.371	0.718	0.884	0.588	0.526

### Inclusion and Exclusion Criteria

Inclusion criteria: ① The preoperative physical examination results met the diagnostic criteria for BPH; ② the patient was willing to undergo conventional PKRP or PKRP with preserved urethral mucosa at the prostatic apex and give written informed consent; ③ the patient had surgical indications; and ④ the postoperative follow-up time was ≥6 months.

Exclusion criteria: ① Patients diagnosed with prostate cancer or who had a history of overactive or neurogenic bladder; ② those with urethral dysfunction caused by bladder calculi, bladder tumor, bladder neck obstruction, preoperative injury of the external urethral sphincter, bladder contracture, and other diseases; ③ those with a history of urethral injury, surgery, and cystotomy; ④ those with urethral stricture and severe weakness of the bladder detrusor induced by other reasons; ⑤ those with changes in the treatment regimen or loss to follow-up; ⑥ those with diabetes for more than 10 years and poor blood glucose control with a history of hypertension and poor blood pressure control normally and sequelae after stroke; ⑦ those with serious diseases of vital organs who likely would be unable to complete the follow-up; and/or ⑧ those with incomplete clinical data.

### Surgical Methods

**Figure 1:**
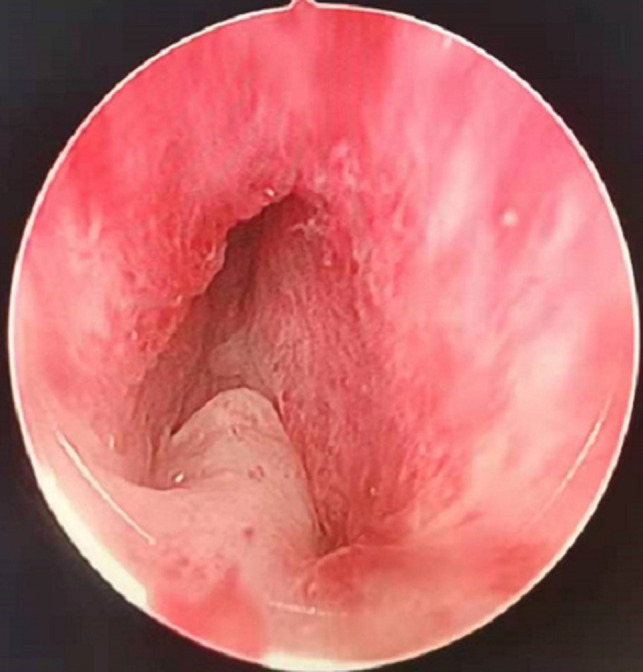
Annular bulge of the urethral mucosa caused by the external urethral sphincter, the prostatic apex protruding into the urethral cavity.

**Figure 2:**
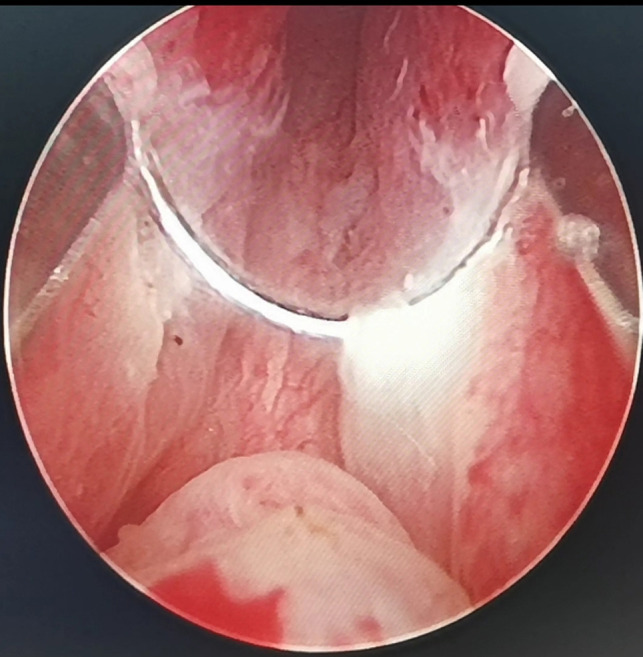
By annularly dissecting the urethral mucosa about 0.5 cm from the apex of the prostate, the urethral mucosa adjacent to the LEEP loop is damaged, whitened and denatured when the electric current is applied.

**Figure 3:**
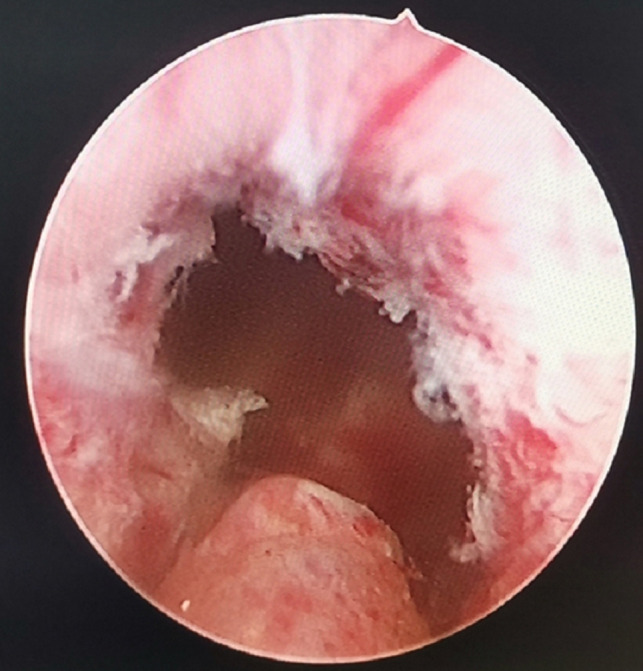
Annular bulge of the urethral mucosa caused by the external urethral sphincter, the preserved urethral mucosa at the prostatic apex.

**Figure 4:**
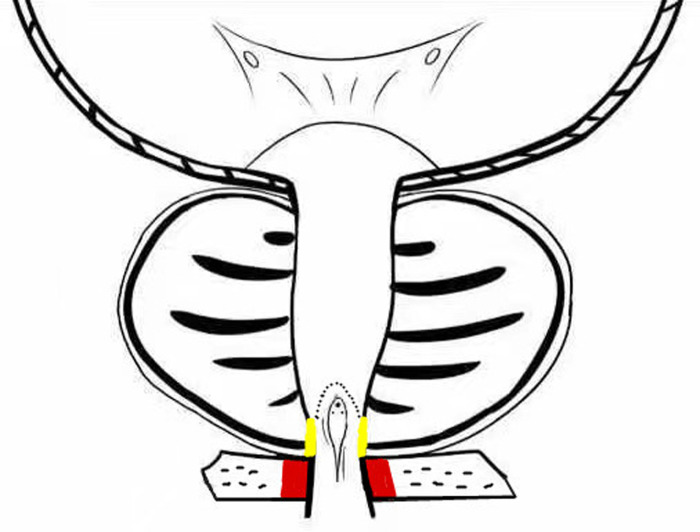
External urethral sphincter (red), preserved urethral mucosa at the prostatic apex (yellow).

The operation was performed with the patient in the lithotomy position under subarachnoid anesthesia, with normal saline as the flushing fluid. The control group was administered conventional PKRP without preserved prostatic urethral mucosa. The observation group was administered PKRP with preserved urethral mucosa at the prostatic apex, as follows: The colliculus was identified as an anatomical landmark, and the hyperplasia of the prostate protruded into the urethral lumen, making it simple to identify the prostatic apex (Figure 1). The urethral mucosa was cut in an annular incision about 0.5 cm from the prostatic apex; the urethral mucosa and the tissue of the prostatic apex were bluntly dissected by LEEP loop (Figure 2), and the dissected urethral mucosa at the prostatic apex was preserved. The prostate tissues were then excised by conventional PKRP. After prostatectomy, the preserved urethral mucosa was trimmed slightly such that the irregular part of the mucosa and a small amount of residual prostate tissues were excised. At the end of the surgery, the external urethral sphincter at the distal end of the urethral mucosa remained intact, and the preserved urethral mucosa at the prostatic apex was located near the urethral sphincter (Figure 3). Figure 4 illustrates the preservation of urethral mucosa at the prostatic apex.

### Outcome Measures

(1) The surgical indicators of the two groups were recorded, including the prostate mass (W*AP*L*0.52*1.05), surgery time, mass of the excised tissue, and glandectomy rate (the mass of the excised prostate tissue/preoperative prostate mass). The intraoperative blood loss results were estimated to be similar to those in other reports (11) (outflow volume of flushing fluid × hemoglobin concentration/preoperative hemoglobin concentration). The grams of excised tissues per unit time were calculated by dividing the mass of excised tissues by the surgery time, which could reflect the speed of the operation.
(2) The PVR and Qmax were tested before surgery, one month after surgery, and three months after surgery, and the patients were instructed to fill in a questionnaire for the IPSS and QoL to evaluate the improvement in symptoms.
(3) The incidences of complications during surgery and three months after surgery in the groups were recorded, including capsular perforation, no prograde ejaculation, urinary incontinence after catheter extraction, electroprostatectomy syndrome, recurrent hematuresis, and postoperative urethrostenosis.

### Statistical Methods

The SPSS 26.0 statistical software was used for analysis. Continuous variables were expressed as the mean ± standard deviation. The t-test was used for the comparison of data between the two groups, and the chi-square test or Fisher’s exact probability method was used for the comparison of the incidence of surgical complications among discontinuous variables. When *P *< 0.05, the difference was considered statistically significant.

## Results

The surgical indicators are shown in Table 2. The resection efficiency was used to reflect the speed of surgery; that of the observation group was significantly higher than that of the control group, with a statistically significant difference (*P *< 0.05). The differences in the glandectomy quality and rate between the groups were not statistically significant (*P *> 0.05), but the surgery time and intraoperative blood loss were significantly lower in the observation group than in the control group, with statistically significant differences (*P *< 0.05).

**Table 2:** Surgical indicators.

Groups	Surgery time /min	Intraoperative blood loss /ml	Grams of prostate excised /g	Glandectomy rate	Surgical resection efficiency
Control group (*n* = 45)	53.87 ± 17.48	85.27 ± 34.06	46.56 ± 18.44	0.72 ± 0.08	0.86 ± 0.20
Observation group (*n* = 45)	44.11 ± 14.18	68.78 ± 27.05	47.62 ± 18.38	0.70 ± 0.10	1.07 ± 0.16
*t*-value	2.907	2.543	−0.275	0.993	−5.29
*P*-value	0.005	0.013	0.784	0.324	<0.001

The postoperative re-examination and follow-up results are shown in Table 3. One month after surgery, the IPSS and QoL in the observation group were lower than those in the control group, with statistically significant differences (*P *< 0.05), while the PVR and Qmax in the two groups were similar, without statistically significant differences (*P *> 0.05). Three months after surgery, the PVR, IPSS, QoL, and Qmax were similar between the groups, with no statistical significance (*P *> 0.05).

**Table 3:** Postoperative re-examination and follow-up results.

Groups	PVR		Qmax		IPSS		Qol	
	One month after surgery	Three months after surgery	One month after surgery	Three months after surgery	One month after surgery	Three months after surgery	One month after surgery	Three months after surgery
Control group (*n* = 45)	9.58 ± 2.93	7.87 ± 1.38	18.32 ± 2.01	19.92 ± 1.30	7.36 ± 1.23	6.51 ± 1.04	1.84 ± 0.67	1.56 ± 0.55
Observation group (*n* = 45)	8.89 ± 2.45	7.51 ± 1.41	18.85 ± 1.24	20.08 ± 1.17	6.49 ± 1.24	6.18 ± 0.94	1.47 ± 0.55	1.51 ± 0.51
*t*-value	1.208	1.212	−1.531	−0.603	3.337	1.601	2.921	0.401
*P*-value	0.230	0.229	0.129	0.548	0.001	0.113	0.004	0.690

The surgical complications of the two groups are shown in Table 4. Immediate urinary continence after catheter extraction was achieved in the observation group. The control group had seven cases of urinary incontinence; among these, four recovered urinary continence within one week after surgery, and the other three recovered it within three months after surgery. The difference between the groups was statistically significant (*P *< 0.05). The incidences of other surgical complications were lower in the observation group than in the control group, and the differences were statistically significant (*P *< 0.05). No TUR syndrome or postoperative urethrostenosis were observed in the groups.

**Table 4:** Surgical complications in the two groups.

Groups	Urinary incontinence after catheter extraction	Other complications					
		Capsular perforation	No anterograde ejaculation	TURS	Urethrostenosis	Recurrent hematuria	Total
Control group (*n* = 45)	7	7	5	0	0	4	16
Observation group (*n* = 45)	0	3	2	0	0	2	7
*χ*^2^-value	–	–	–	–	–	–	4.731
*P*-value	0.012	–	–	–	–	–	0.030

## Discussion

The results of this study showed that PKRP with preserved urethral mucosa at the prostatic apex can significantly reduce the probability of urinary incontinence compared with conventional PKRP and lead to immediate urinary continence after catheter extraction, with higher glandectomy efficiency and less intraoperative blood loss. Moreover, although the urethral mucosa at the prostatic apex was preserved, there was no significant difference in the glandectomy rate between the observation and control groups, which did not affect the excision of hyperplastic glands. The observation group had fewer intraoperative and postoperative complications than the control group, and the short-term IPSS and QoL in the observation group were better. The indexes of the two groups were similar at about three months after surgery, showing the similar long-term clinical efficacy of the different surgical methods. However, this study had a small sample size and short follow-up time, so studies with longer follow-up time and larger sample size are required to validate these results.

In the novel surgical method, the urethral mucosa at the prostatic apex was preserved in a way similar to open prostatectomy. Additionally, in the improved surgery, an efficacy similar to that of open prostatectomy could be achieved, and the benefits of TURP could be maintained to benefit the patients. Compared with conventional PKRP, this method has the following advantages:

### It Helps Protect the External Urethral Sphincter, as Follows

(1) External urethral sphincter injury leads to urinary incontinence (12), and identification of the sphincter is one of the difficulties of TURP. Since the external urethral sphincter surrounds the outside of the membranous urethra, it cannot be observed directly. During surgery, it is located indirectly by observing the annular bulge of the urethral mucosa at the distal end of the seminal colliculus (Figure 1). When the prostate is hyperplastic and the surgery time is long, the repeated intrusion of the endoscope will cause hemorrhaging and erosion of the urethral mucosa, increasing the difficulty of identifying the external urethral sphincter (Figure 3). The PKRP with preserved urethral mucosa at the prostatic apex solves this problem simply and effectively. To start, the prostatic apex bulging into the urethral cavity is easily identified (Figure 1). By simple incision and dissection, the urethral mucosa of the prostatic apex is preserved. At this time, the external urethral sphincter must be located about 0.5 cm from the distal end of the urethral mucosa incision line (Figure 2). During the subsequent surgery, the external urethral sphincter is fully protected by the urethral mucosa at the prostatic apex, which has already been dissected and thus is easily identified.
(2) To avoid the “bottleneck effect,” excision of the prostatic apex is a vital operation in the late stage of surgery (13, 14) and the most important factor in avoiding external urethral sphincter injury. At this point, the surgeon is expected to excise the prostate tissues as thoroughly as possible to allow the patients to void with a good stream after surgery and to avoid external urethral sphincter injury to prevent postoperative urinary incontinence. This process is often difficult and tiring for the surgeon. Moreover, the surgeon’s energy has decreased significantly by the late stage of surgery, which increases the chance of injury to the external urethral sphincter. In PKRP with preserved urethral mucosa at the prostatic apex, the external urethral sphincter is basically protected at the beginning of the surgery, when the surgeon is still energetic, and the excision of hyperplastic tissues at the apex is also made easier, which significantly reduces the surgeon’s energy consumption and makes the surgery simpler.
(3) It was also observed that the electric current could cause damage to adjacent tissues, as shown in Figure 2. When the LEEP loop is energized, the adjacent urethral mucosa can be damaged, whitened, and denatured. The urethral mucosa of the prostatic apex is not preserved in conventional PKRP, and the electric current will inevitably cause injury to the adjacent external urethral sphincter during excision of the prostatic apex. In the new surgery, the preserved urethral mucosa at the prostatic apex is located between the LEEP loop and the external urethral sphincter, acting as a spacer when the electric current is applied. The electric current may damage the preserved urethral mucosa but not the external urethral sphincter (Figure 2).

### The Preserved Urethral Mucosa at the Prostatic Apex Acts as a Sealing Pad

Zinner found that the urethral mucosa plays an important role in urinary continence (15). Compared with conventional PKRP, the method of artificially preserving the urethral mucosa at the prostatic apex increases the length of the urethral mucosa and the number of folds near the external urethral sphincter and been lined on the external urethral sphincter like a sealing pad, which can help the external urethral sphincter increase the urethral closure pressure for better closure and facilitate postoperative urinary continence.

### The Novel Method can also Improve the Efficiency of Prostatectomy

Once identified at the beginning of the surgery, the preserved urethral mucosa at the prostatic apex can be used as an artificial anatomical marker of the endpoint for prostatectomy. The dissected urethral mucosa can be easily observed. Before the preserved urethral mucosa, the prostate should be excised safely and quickly. It is easily controlled throughout the surgery without needing to identify the external urethral sphincter, which can significantly shorten the surgery time, reduce blood loss, improve the efficiency of resection, and minimize the surgeon’s fatigue and the surgical risks (Table 4).

In this study, in the early stage of PKRP with preserved urethral mucosa at the prostatic apex, two patients had dysuria after catheter extraction, which may be ascribed to the overlong preservation of the urethral mucosa. The catheter indwelling time for open prostatectomy was referred to, and the catheter was indwelled for 12 days. The patients urinated normally after catheter extraction, which may be related to atrophy of the preserved urethral mucosa and the urethral mucosa after wound healing or injury in prostatectomy (no longer obstructing the urinary flow). As with conventional PKRP, the urethra was cut into a lambdoidal suture at points 5–7 to keep the urinary tract unobstructed (Figure 3). Before the end of the surgery, the resectoscope was pulled out after the bladder was filled, and the bladder region was compressed to allow for smooth urine flow to help determine the surgical effect. If bradyuria occurred, it could be ascribed to the overlong preservation of the urethral mucosa; in this case, part of the long urethral mucosa would be excised. There were no other cases of dysuria after catheter extraction.

## Conclusion

Transurethral PKRP with preserved urethral mucosa at the prostatic apex is a safe and simple operation and an improvement of the minimally invasive surgical technique for BPH. Compared with conventional PKRP, it can effectively lead to immediate postoperative urinary continence, significantly reducing the surgeon’s concerns about intraoperative and postoperative urinary incontinence. Moreover, it can improve the surgical efficiency, reduce the surgical difficulty, and shorten the learning curve of PKRP. Thus, it is worth promoting in clinical application. However, this study had a small sample size and short follow-up time, so studies with a longer follow-up time and larger sample size are required to validate these results.

## Data Availability

The original contributions presented in the study are included in the article/Supplementary Material, further inquiries can be directed to the corresponding author/s.
